# WEDM: Wavelet-Enhanced Diffusion with Multi-Stage Frequency Learning for Underwater Image Enhancement

**DOI:** 10.3390/jimaging11040114

**Published:** 2025-04-09

**Authors:** Junhao Chen, Sichao Ye, Xiong Ouyang, Jiayan Zhuang

**Affiliations:** 1 Faculty of Mechanical Engineering & Mechanics, Ningbo University, Ningbo 315211, China; chenjunhao@nimte.ac.cn; 2 Ningbo Institute of Materials Technology & Engineering, Chinese Academy of Sciences, Ningbo 315201, China; yesichao@nimte.ac.cn; 3 Faculty of Electrical Engineering and Computer Science, Ningbo University, Ningbo 315211, China; ouyangxiong@nimte.ac.cn

**Keywords:** underwater image enhancement, wavelet transform, frequency-domain processing, diffusion models

## Abstract

Underwater image enhancement (UIE) is inherently challenging due to complex degradation effects such as light absorption and scattering, which result in color distortion and a loss of fine details. Most existing methods focus on spatial-domain processing, often neglecting the frequency-domain characteristics that are crucial for effectively restoring textures and edges. In this paper, we propose a novel UIE framework, the Wavelet-based Enhancement Diffusion Model (WEDM), which integrates frequency-domain decomposition with diffusion models. The WEDM consists of two main modules: the Wavelet Color Compensation Module (WCCM) for color correction in the LAB space using discrete wavelet transform, and the Wavelet Diffusion Module (WDM), which replaces traditional convolutions with wavelet-based operations to preserve multi-scale frequency features. By combining residual denoising diffusion with frequency-specific processing, the WEDM effectively reduces noise amplification and high-frequency blurring. Ablation studies further demonstrate the essential roles of the WCCM and WDM in improving color fidelity and texture details. Our framework offers a robust solution for underwater visual tasks, with promising applications in marine exploration and ecological monitoring.

## 1. Introduction

As humanity advances in the exploration and utilization of marine resources, underwater imaging technology has become increasingly critical in domains such as marine biology monitoring and seabed resource exploration [1,2,3]. However, due to the absorption and scattering properties of water, underwater images often suffer from severe degradation, including color distortion, loss of structural detail, and noise interference [4]. These degradations not only impair visual perception but also significantly hinder downstream analysis tasks, such as detection, segmentation, and tracking [5]. Consequently, underwater image enhancement (UIE) aims to restore visual fidelity by compensating for scattering effects and correcting color shifts, thereby improving the utility of underwater images in diverse computer vision applications [6].

To enhance the quality of underwater images, researchers have developed both traditional and deep learning-based approaches. Traditional UIE methods typically rely on prior knowledge, heuristic assumptions, or simplified physical models, such as histogram equalization [7] and white balance correction [8]. While these techniques can partially improve image contrast and color balance, their effectiveness is inherently constrained by their reliance on overly simplistic assumptions that fail to capture the complex optical properties of underwater environments. Consequently, when applied to real-world underwater scenes, traditional methods often introduce visual artifacts and color inaccuracies. Moreover, they generally disregard frequency-domain characteristics, resulting in suboptimal preservation of fine details and structural information [9].

In recent years, deep learning-based UIE methods have garnered significant attention. These methods leverage convolutional neural networks (CNNs) or generative adversarial networks (GANs) to learn the mapping between degraded and clear images from extensive datasets, significantly enhancing image quality. However, most CNN-based methods adopt direct regression strategies. Although these streamline the processing pipeline and enhance clarity and contrast, they generate deterministic images, lacking flexibility and diversity [10]. Consequently, they often exhibit inconsistent performance in complex underwater scenarios and struggle to adapt to various water bodies, lighting conditions, and degradation factors. In contrast, GAN-based methods employ adversarial training to address stability issues and generate more diverse and realistic images [11]. However, GANs often face challenges such as mode collapse and gradient vanishing during training, limiting their widespread application in UIE tasks.

With increasing interest in generative models, diffusion-based models [12,13] have emerged as a prominent research focus, demonstrating exceptional performance in the generation of high-quality and diverse images. These models have gradually become the state-of-the-art (SOTA) benchmark in image generation. Through a gradual denoising process, diffusion models effectively capture the uncertainty and diversity inherent in UIE image generation, overcoming the limitations of GANs in terms of training stability and mode diversity. Compared to GANs, diffusion models are more robust, yielding finer and more realistic images while avoiding the risk of mode collapse. Nevertheless, most existing diffusion models focus on global feature modeling in the spatial domain, neglecting the processing of frequency-domain information and fine-grained details. In high-noise underwater environments, traditional diffusion models struggle to restore high-frequency details and may amplify noise during denoising, ultimately diminishing the quality of generated images [14].

**Figure 1:**
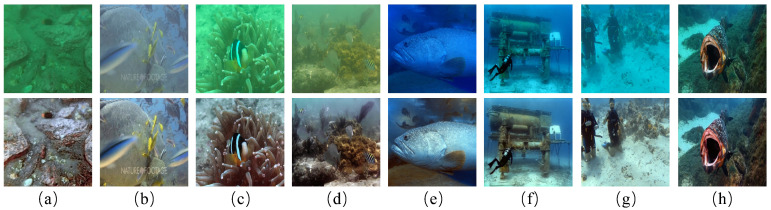
Enhanced results (bottom) of our WEDM for several raw images (top). (**a**,**b**) are derived from the LSUI [15], (**c**,**d**) are derived from the UIEB [16], (**e**,**f**) are derived from the U45 [17], and (**g**,**h**) are derived from the Challenge60. Our WEDM obtained satisfactory visual results for different degraded images from different datasets.

To address these challenges and enhance underwater image quality, we propose a novel UIE framework based on the Conditional Residual Denoising Diffusion Model (RDDM), named the WEDM. This framework combines frequency domain information with the attributes of diffusion models to generate high-quality and diverse samples through a conditional guidance mechanism. Specifically, the WEDM framework enhances images in two stages. In the first stage, we introduce the Wavelet Color Compensation Module (WCCM), which uses discrete wavelet transform (DWT) to decompose the image into low- and high-frequency components for enhancement. This module compensates for color distortion and detail loss in underwater images, with the enhanced image serving as a conditional input for the subsequent generation process. In the second stage, we present the Wavelet Diffusion Module (WDM). The WDM replaces traditional convolution operations with wavelet convolution (WTConv) and integrates this module deeply into the U-Net structure of the diffusion model. This approach strengthens the ability of the model to restore details and textures of the image. The WDM uses WTConv to extract multiscale features in the frequency domain, effectively preventing the oversmoothing of high-frequency details during denoising, a common issue in traditional diffusion models. Our method combines the powerful feature representation of diffusion models with the precision of frequency-domain processing, improving noise robustness and significantly enhancing image enhancement results and generalization ability. In addition, Figure 1 demonstrates the enhancement results of the degraded images from different datasets by the WEDM.

In summary, the main contributions of our method are as follows:

- We propose a novel UIE framework, the WEDM, based on WTConv and diffusion models. This framework addresses the frequency domain characteristics of underwater images and the degradation mechanism of diffusion models in high-frequency information, successfully implementing frequency domain enhancement and diffusion adjustment. This significantly improves the model’s ability to restore underwater image details and textures.
- We introduce the Wavelet Color Compensation Module (WCCM), which uses discrete wavelet transform for frequency domain decomposition and enhancement fusion. This effectively compensates for the degradation in underwater images. In the WEDM, the image processed by the color compensation module serves as a strong conditional guide, driving the diffusion model’s denoising process to accurately restore high-frequency details and reduce recovery bias caused by degradation.
- We propose the WTConv Residual Diffusion Adjustment Module (WDM), which deeply explores the potential of diffusion models in frequency domain modeling. This significantly improves the restoration of image details and textures while enhancing the model’s robustness to noise and generalization capabilities.
- Experimental results show that the WEDM outperforms previous UIE methods. Extensive ablation experiments validate the effectiveness of our contributions.

## 2. Related Work

### 2.1. Traditional Underwater Image Enhancement Method

Traditional UIE methods are predominantly based on either physical models or image enhancement strategies. Physical model-based methods aim to reverse the degradation process by constructing estimation models for underwater imaging parameters such as attenuation coefficients and background light. Peng et al. [18] improved the dark channel prior to separate scattering light and perform color correction. However, these methods are vulnerable to color distortion, particularly due to errors in depth map estimation in complex scenes. Liang et al. [19] proposed a multi-constraint optimization model that incorporates priors such as the gray world assumption. However, these models are highly sensitive to non-uniform illumination in underwater environments. While these methods are physically interpretable, their reliance on manually specified priors limits their ability to adapt to the dynamic and complex optical conditions encountered underwater, often resulting in artifacts and local over-enhancement.

In contrast, image enhancement methods work by improving visual quality through pixel-level operations. For instance, the Retinex fusion method proposed by Zhang et al. [20] improves contrast but exacerbates high-frequency noise. Ancuti et al. [21] implemented a multi-strategy fusion approach to achieve color balance, but their method neglects the wavelength-dependent nature of underwater scattering, leading to imbalance in channel compensation. It is crucial to note that existing enhancement techniques face a trade-off: global operations such as histogram equalization [22] may damage local details, whereas block-based variational methods [23] can enhance texture but introduce block effects. This disconnect between low-level signal processing and high-level semantic understanding hinders the practical application of traditional methods in real underwater scenarios.

### 2.2. Deep Learning Underwater Image Enhancement Methods

With the rise of deep learning, data-driven UIE methods have increasingly become the standard approach. These methods capitalize on the powerful feature representation and non-linear mapping capabilities of neural networks, which automatically extract relevant features from training data. CNN architectures model degradation patterns through end-to-end mappings. For instance, UWCNN [24] adopts a multi-branch architecture to adapt to varying water qualities, while Ucolor enhances discriminability by fusing features across different color spaces. However, deterministic regression frameworks often lead to local optima, resulting in limited output diversity and instability, especially in scenarios involving extreme degradation. To address these limitations, adversarial generation mechanisms have been introduced. Water-Net [25] developed a multi-task GAN framework for joint dehazing and color restoration, while CycleGAN-based methods [26,27] tackle the issue of data scarcity through unsupervised domain adaptation. While GANs excel at generating fine details, the dynamic interplay between the discriminator and the generator can lead to mode collapse, producing structural artifacts in the generated images.

In recent years, several complex frameworks have been proposed and have achieved state-of-the-art performance [28,29]. For instance, Yang et al. [30] proposed a multi-scale progressive restoration network that is aware of reflected light. This network is capable of producing images with both color equalization and rich texture in a variety of underwater scenes. Huang et al. [31] proposed a mean teacher-based semi-supervised network, which effectively leverages the knowledge from unlabeled data. Recent studies suggest that current methods predominantly focus on spatial domain feature learning, whereas the low-frequency energy decay and high-frequency phase distortion characteristics of underwater images in the frequency domain remain underexplored [32], which represents a critical bottleneck limiting the improvement of model performance.

### 2.3. Diffusion Models

**Figure 2:**
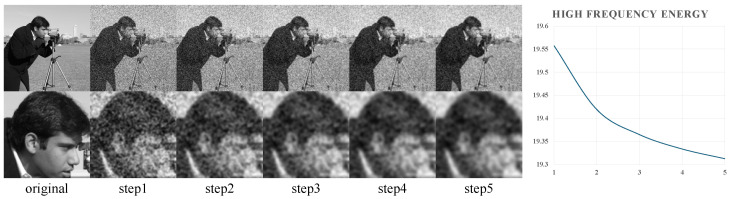
Decay process of high-frequency details under multiple noise injections. (**Left**) This section illustrates the evolution of the image after noise is added. Gaussian blur is used to simulate intermediate states during the diffusion process. As noise gradually increases, the high-frequency details of the image become progressively blurred and eventually disappear. (**Right**) This graph shows the trend of high-frequency energy in the image. As noise is gradually injected, the high-frequency energy continuously decays, reflecting the gradual disappearance of high-frequency details.

Diffusion models have achieved significant breakthroughs in generation quality and diversity by utilizing a progressive denoising mechanism, offering a novel paradigm for UIE. Saharia et al. [33] proposed Palette, which has demonstrated the excellent performance of diffusion models in the field of conditional image generation, including colorization, in-painting, and JPEG restoration. Ref. [34] were the first to apply this approach to underwater image enhancement, restoring details through iterative noise correction. However, traditional diffusion models exhibit two primary limitations: first, high-frequency details tend to be smoothed out during multiple noise injections (as shown in Figure 2); second, global denoising strategies struggle to distinguish between signal and complex underwater noise, which leads to local contrast imbalances.

Recent studies have begun to explore frequency-domain-guided diffusion mechanisms. WF-Diff [35] employs wavelet decomposition for multi-scale generation, but directly applying this approach to UIE tasks introduces issues related to color channel coupling. Therefore, there is a pressing need to design a collaborative enhancement framework that combines frequency-domain and spatial-domain features, tailored to the specific characteristics of underwater imaging.

## 3. Methodology

### 3.1. Overall Framework

**Figure 3:**
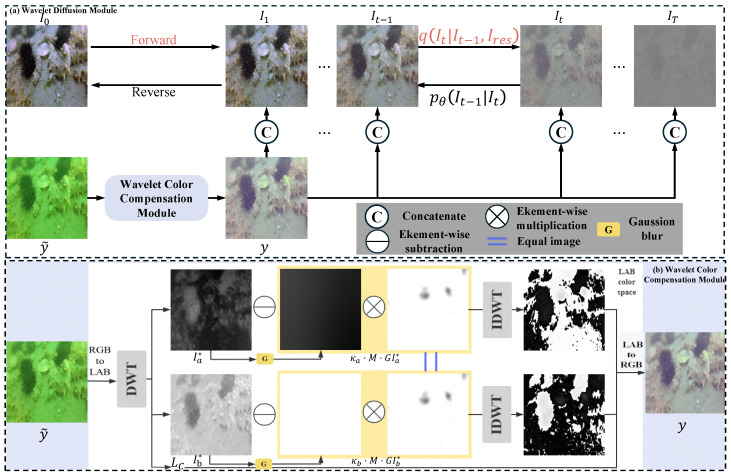
Architecture of the proposed WEDM method. It contains two detachable networks, the Wavelet Color Compensation Module (WCCM) and the Wavelet Diffusion Module (WDM). The degraded underwater image $\tilde{y}$ obtains the conditional image *y* through the WCCM.

This study is dedicated to developing a network capable of concurrently eliminating color bias and enhancing the details of underwater images. The proposed Wavelet-based Underwater Image Enhancement Diffusion Model (WEDM), as depicted in Figure 3, seamlessly integrates the advantages of frequency-domain information with the formidable capabilities of diffusion models.

The proposed WEDM framework comprises two synergistic modules: the Wavelet Color Compensation Module (WCCM) for color restoration and the Wavelet Diffusion Module (WDM) for detail enhancement. Underwater images typically suffer from frequency-dependent degradations; for example, global color casts and scattering predominantly affect low-frequency components, whereas detail blurring manifests as high-frequency information loss. To address this, the WCCM applies discrete wavelet transform (DWT) to decompose the input image into sub-bands. Through LAB color space transformation and frequency-domain operations, it generates a pre-enhanced conditional image that corrects low-frequency color distortion while retaining high-frequency structure.

**Figure 4:**
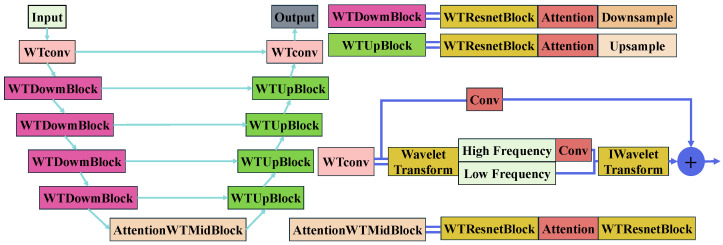
Description of the U-Net architecture with skip connections. The architecture consists of downsampling and upsampling paths, incorporating wavelet convolutions and residual blocks for feature extraction. The intermediate stage integrates an attention mechanism to capture long-range dependencies and enhance feature extraction.

The WDM adopts the RDDM network [13] as its denoising backbone. As illustrated in Figure 4, it utilizes a U-Net-like architecture where traditional convolution is replaced with WTConv. This enables effective multi-scale feature extraction in the frequency domain. The residual diffusion process enhances high-frequency components while avoiding the over-smoothing typical in conventional models. Overall, the cooperative design of the WCCM and WDM ensures both global structure recovery and fine detail preservation, significantly boosting enhancement performance under complex underwater degradation scenarios.

To justify the use of wavelet transform, we begin by modeling the physical characteristics of underwater imaging. These images often suffer from scattering and absorption effects, which can be modeled as:

(1) $$I_{in}=\alpha I_{clean}+\beta I_{distortions}$$

where $I_{clean}$ denotes the latent clean image, $I_{distortions}$ represents distortions caused by the underwater environment, and α, β are coefficients determined by water’s optical properties.

Next, discrete wavelet transform (DWT) is applied to decompose the image into frequency-specific components:

(2) $$I_{LL},\left\{I_{LH},I_{HL},I_{HH}\right\}=DWT\left(I\right)$$

where, $I_{LL}$ contains low-frequency components typically associated with color degradation, while $\left\{I_{LH},I_{HL},I_{HH}\right\}$ capture high-frequency details susceptible to blur. The biorthogonal nature of DWT ensures lossless decomposition, enabling degradation-specific processing in the frequency domain.

In contrast, standard convolutions process all frequencies jointly, leading to frequency aliasing and mixing of structural and detail information. This often results in blurred textures and edge loss.

To overcome this, wavelet convolution (WTConv) is employed to independently process each sub-band, effectively decoupling the optimization objectives.

From the perspective of diffusion modeling, denoising is formulated as a conditional probability process:

(3) $$q\left(x_{t-1}|x_{t},x_{0}\right)=N\left(x_{t-1};\tilde{\mu }\left(x_{t},x_{0}\right),{\tilde{\beta }}_{t}I\right)$$

Integrating WTConv into the diffusion framework enables sub-band-specific noise prediction. This reduces problem complexity, constrains the solution space, and enhances prediction accuracy. By guiding denoising in separate frequency domains, the model avoids high-frequency detail loss and improves robustness. Recent studies [36] have demonstrated the effectiveness of similar frequency-guided strategies, further validating our approach.

### 3.2. Wavelet Color Compensation Module

The purpose of the WCCM module is to perform color correction on the low-frequency components and enhance high-frequency details of the underwater image through frequency-domain operations. The steps involved are as follows:

1. LAB conversion: Convert the RGB image to the LAB color space, which allows for the separation of luminance (*L*) and chrominance (*a*, *b*) channels. This separation facilitates the independent adjustment of color components, where the luminance information is less affected by the scattering effects of water.
2. Mask generation: Based on the luminance values, a binary mask M(x) is generated using a thresholding approach. Specifically, pixels with luminance L(x) greater than 0.847 are assigned a value of zero, and others are assigned one. This mask enables discriminative processing of regions with higher and lower luminance, ensuring that color compensation is applied appropriately based on the image’s luminance distribution.
  (4) $$M\left(x\right)=I\left(L(x)<0.847\right)\cdot G\left(\sigma =1.5\right)$$
  where G(σ=1.5) represents a Gaussian blur applied to the mask for smoothing purposes. The threshold value of 0.847 is determined based on the Color Channel Compensation (3C) method [37], which experimentally shows that luminance values above 0.85 usually correspond to strong light sources or overexposed regions. Such areas are unsuitable for aggressive color correction, as it may introduce additional distortion. Considering the unique luminance distribution of underwater images, we slightly adjusted this threshold to 0.847 to better suit underwater scenes.
3. Wavelet-based correction: After decomposition of the image into wavelet subbands, color correction is applied to the low-frequency subbands of the *a* and *b* chrominance channels. This step adjusts for the color bias introduced by the underwater environment.
  (5) $$W{\left(I_{c}\right)}_{LL}\leftarrow W{\left(I_{c}\right)}_{LL}-\kappa_{c}\cdot M\cdot G\left(W{\left(I_{c}\right)}_{LL}\right)$$
  where c∈{a,b} and $\kappa_{c}$ controls the strength of color correction. The Gaussian-blurred mask *M* is used to modify the low-frequency components of the chrominance channels, helping to mitigate color distortion while preserving essential image structures. The parameter $\kappa_{c}$ is set with reference to the 3C method [37], which demonstrates that moderate values yield optimal results in standard imaging conditions. In underwater scenarios, where the red channel often experiences severe attenuation, increasing $\kappa_{c}$ to around 1.0 significantly improves compensation. This study follows the empirical guideline while adjusting $\kappa_{c}$ for underwater characteristics.
4. Reconstruction: After color correction is applied to the low-frequency subbands, the image is reconstructed by merging all subbands using the inverse discrete wavelet transform (IDWT). The final image is then converted back to the RGB color space to obtain the color-corrected result.

By working in the wavelet domain, this module allows for fine-grained control over color compensation while maintaining the integrity of the image’s structural information.

### 3.3. Wavelet Diffusion Module

The objective of the WDM is to combine the advantages of multi-scale feature extraction in the wavelet domain with the strong representation capabilities of diffusion models to refine the image’s high-frequency and low-frequency components. The WDM performs both forward and reverse diffusion processes, detailed as follows:

1. Forward Diffusion: The forward diffusion process is modeled as a Markov chain, where noise is gradually added to the image. The forward step for a single noise addition can be written as
  (6) $$q\left(I_{t}|I_{t-1},I_{res}\right)=N\left(I_{t};I_{t-1}+\alpha_{t}I_{res},\beta_{t}^{2}I\right)$$
  Here, $I_{t}$ represents the image at time step *t*, and $I_{res}=I_{in}-I_{0}$ is the residual between the degraded image $I_{in}$ and the clean image $I_{0}$. The parameters $\alpha_{t}$ and $\beta_{t}$ control the influence of the residual and the noise respectively.
2. Wavelet Convolution Parameter Setting and Sensitivity Analysis: The WTConv module leverages wavelet transform to effectively avoid the frequency aliasing problem inherent in conventional convolution, while achieving a large receptive field without significantly increasing computational cost. We adopt the db1 wavelet (Haar wavelet), which provides clear frequency localization and minimal computational complexity in image processing tasks. In addition, the wavelet convolution kernel size is set to 5 × 5. Experimental results show that, compared with smaller kernels, the 5 × 5 kernel is more effective in capturing long-range dependencies among image features, thereby better preserving and restoring high-frequency details. Following the experimental study of Finder et al. [38], we set the wavelet decomposition level to 2, which yields superior multi-scale feature representations.
3. Reverse Denoising: The reverse diffusion employs L1 loss for residual prediction to preserve high-frequency details. The reverse denoising process is modeled as follows:
  (7) $$p_{\theta }\left(I_{t-1}|I_{t},I_{in}\right)=N\left(I_{t-1};\mu_{\theta }\left(I_{t},I_{in},t\right),\sigma_{t}^{2}I\right)$$
  where $I_{in}$ is the condition image, and $\mu_{\theta }\left(I_{t},I_{in},t\right)$ represents the predicted mean at step *t*. The variance $\sigma_{t}^{2}$ is calculated based on the previous time step. The network uses this mean and variance to predict the denoised residuals.
4. Feature Reconstruction: After the WTConv operations, the processed high-frequency subbands are scaled to control their influence. The feature map is then reconstructed by merging the high-frequency and low-frequency components and applying inverse wavelet transform (IWT):
  (8) $$\hat{X}=I_{WT}\left(\left\{X_{LL},{\hat{X}}_{LH},{\hat{X}}_{HL},{\hat{X}}_{HH}\right\}\right)$$
  due to the biorthogonal property of DWT, the reconstructed feature map is then added as a residual to the original input feature map, ensuring that the details and structures are preserved without causing distortion:
  (9) $$X_{out}=X+\alpha \cdot \hat{X}$$
  where α is a coefficient that controls the influence of the residual, typically set to 0.1 to prevent over-adjustment.
5. Training Process and Loss Function: During the reverse diffusion process, the model predicts both the residuals $I_{res}$ and the noise ϵ. The training objective is to minimize the Kullback–Leibler (KL) divergence between the true posterior and the predicted posterior, which is simplified to the following L1 loss:
  (10) $$L\left(\theta \right)=E_{\left(t,I_{t},I_{res}\right)}\left[∥I_{res}-I_{res}^{\theta }\left(I_{t},t\right){∥}_{1}\right]$$
  This simplified loss function ensures that the model learns to predict the residuals effectively, leading to better performance in underwater image enhancement tasks.

## 4. Experiments

### 4.1. Setup

**Implementation Details.** The proposed WEDM method was trained on an NVIDIA RTX A5000 GPU with 1000 diffusion steps. The Adam optimizer and L1 loss function were used for a total of 240,000 iterations. The initial learning rate was set to 8 × $10^{-5}$, and the batch size was fixed at 8. The training dataset consisted of 3900 randomly selected underwater image pairs from the LSUI dataset [15], which were cropped to 256 × 256 pixels before being fed into the network. During inference, full-resolution testing was performed using three diffusion steps for all tasks.

**Datasets.** The WEDM model was trained on 3900 randomly selected image pairs from the LSUI dataset. The remaining 379 images were set aside as the test set (referred to as TEST-L400). Additionally, the generalization performance of the WEDM was evaluated on real-world underwater scenes from the UIEB dataset [16], the Challenge60 dataset, and the U45 dataset [17].

**Comparison Methods.** We compared the WEDM with seven SOTA UIE methods, which include one image restoration method (UDCP [39]), one image enhancement method (WWPF [40]), and five deep learning-based methods (PUIE-Net [41], FUnIE-GAN [29], LENet [42], Shallow-UWnet [43], and DM-water [34]). Among these, DM-water is a diffusion-model-based method. To ensure a fair and rigorous comparison, we used the source code provided by the respective authors and adhered to the same experimental settings for evaluation.

**Evaluation Metrics.** To assess the performance of different UIE methods, we used full-reference image quality metrics, PSNR [44] and SSIM [45], to quantify the enhancement effects of the WEDM on the LSUI and UIEB datasets. These metrics were computed based on the Y channel in the YCbCr color space. Additionally, we used non-reference image quality metrics, UIQM [46] and UCIQE [47], to evaluate the performance of the model on non-reference benchmarks such as the U45 and Challenge60 datasets.

### 4.2. Results and Comparisons

#### 4.2.1. Qualitative Comparison

For datasets with reference data, we compared the WEDM with other methods on the TEST-L400 and UIEB datasets to evaluate its effectiveness in natural underwater image enhancement. The results are presented in Table 1. Compared to existing methods, the WEDM demonstrates a clear advantage in preserving the structural information and details of the enhanced images, achieving the highest PSNR and SSIM scores. Relative to the second-best method, the WEDM shows an 18.33% improvement in PSNR and a 1.58% increase in SSIM. The WEDM method emphasizes preserving image structure and texture details rather than merely minimizing mean squared error, highlighting its effectiveness in enhancing underwater images that are sensitive to structural details. This further confirms the robustness of the proposed WEDM method.

**Figure 5:**
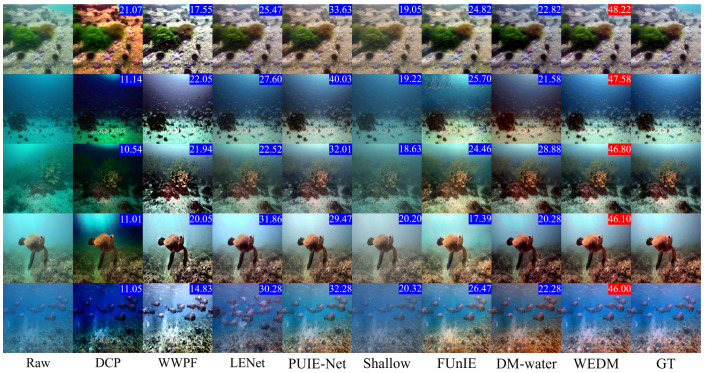
Visual Comparison of real underwater images. Five original images (left column) are drawn from the TEST-L400 subset of the LSUI dataset. The PSNR metric is displayed in the upper right corner, with the best score highlighted in red.

**Figure 6:**
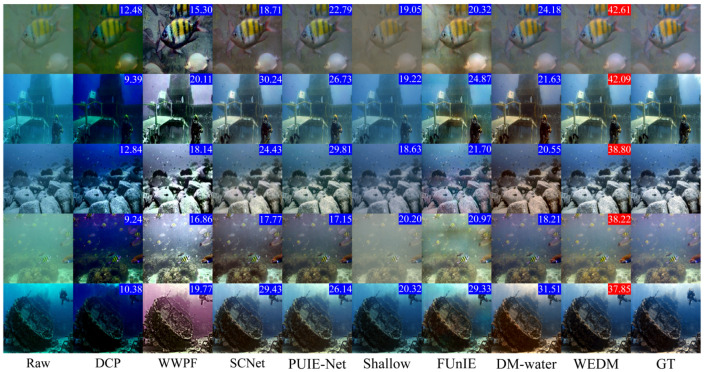
Visual comparison of real underwater images. Five original images (left column) are drawn from the UIEB dataset. The PSNR metric is indicated in the upper right corner, with the best value highlighted in red.

Additionally, Figure 5 and Figure 6 present visual comparisons of images enhanced by the WEDM and other methods across different degraded underwater scenes. The experimental results show that FUnIE-GAN and Shallow-UWnet underperform in enhancing degraded images due to limited parameters, with FUnIE-GAN introducing noticeable noise. PUIE-NET and LENet improve image clarity but suffer from color distortion, and the enhanced images require further adjustment of saturation and contrast. DM-water achieves good results in detail enhancement but still requires improvement in color quality. WWPF produces enhanced images with good color quality and contrast by adjusting contrast and sharpening, but it also leads to unnatural artifacts, loss of details, and amplified noise. The visual enhancement results in Figure 5 and Figure 6 demonstrate the effectiveness of the WEDM in enhancing degraded underwater images across various scenarios. Compared to other methods, the WEDM shows significant advantages in restoring image details, color, and contrast. Moreover, while some comparison methods perform better on hazy or blue-tinted images, none provide satisfactory results across all scenarios. The WEDM demonstrates strong generalization capabilities, maintaining image structure and texture details, with processed images closely resembling reference images and achieving realistic restoration. Therefore, based on both qualitative and quantitative metrics, the WEDM outperforms current SOTA models.

On one hand, underwater imaging is an ill-posed problem, meaning that restoration outcomes are not unique, and no definitive ground truth exists. On the other hand, non-reference image quality assessment (NR-IQA) better reflects human visual perception compared to full-reference IQA (FR-IQA). Therefore, we tested the WEDM on non-reference datasets (Challenge60 and U45) and used NR-IQA metrics to quantify the enhancement results, demonstrating the WEDM’s superiority. The results are shown in Table 2.

**Figure 7:**
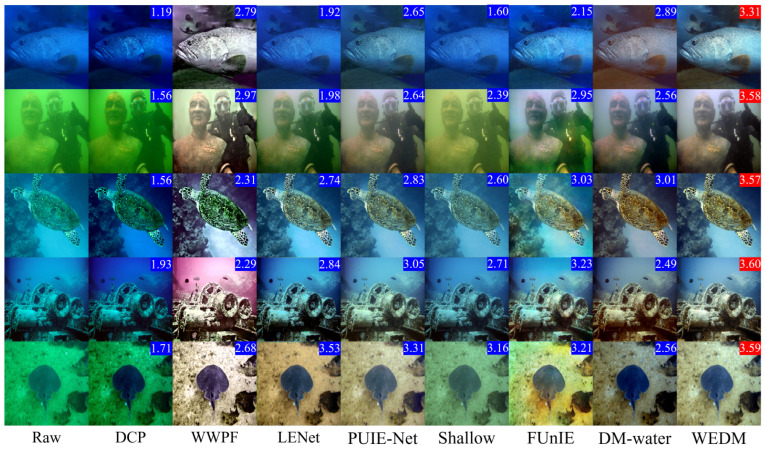
Visual Comparison of real underwater images. Five original images (left column) are from the U45 dataset. The UIQM metric is indicated in the upper right corner, with the best value highlighted in red.

**Figure 8:**
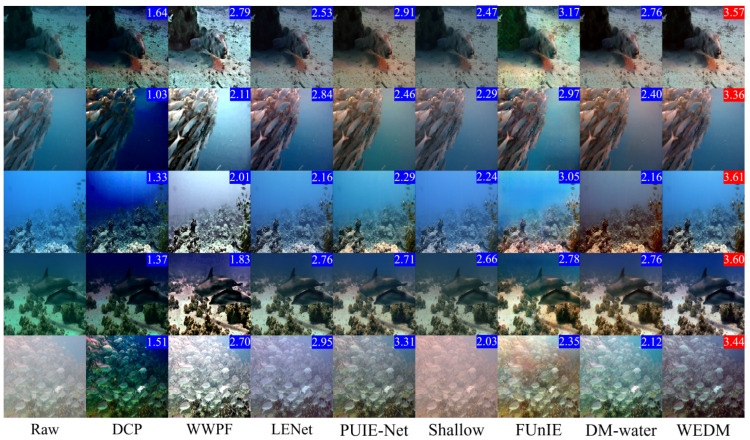
Visual comparison of real visual comparison of real underwater images. Five original images (left column) are drawn from the Challenge60 dataset. The UIQM metric is displayed in the upper right corner, with the best value highlighted in red.

Table 2 demonstrates that the WEDM achieves the highest UIQM and UCIQE scores on every dataset. Evaluations on the Challenge60 and U45 datasets show that the WEDM performs exceptionally well on both UIQM and UCIQE metrics, highlighting its significant advantages in enhancing image quality and restoring accurate colors. These results further validate the effectiveness and feasibility of the WEDM method for UIE tasks. Figure 7 and Figure 8 present visual comparisons of these datasets. The DCP method improves image contrast by introducing artificial colors and reducing blur, but the enhanced images exhibit darker tones and unrealistic appearances. The WWPF method uses a locally adaptive strategy to improve image quality, but its performance is limited by underwater imaging characteristics and cannot achieve optimal enhancement under certain conditions. The Shallow method partially restores details but has limited enhancement, often introducing red shift when processing turbid, complex underwater images. PUIE-Net exhibits limited generalization across different underwater environments, resulting in unnatural color reproduction. In contrast, the WEDM delivers superior underwater image restoration quality, with better clarity, contrast, color fidelity, and naturalness. It achieves consistent restoration of color and detail that aligns with real underwater environments and meets human visual standards.

**Figure 9:**
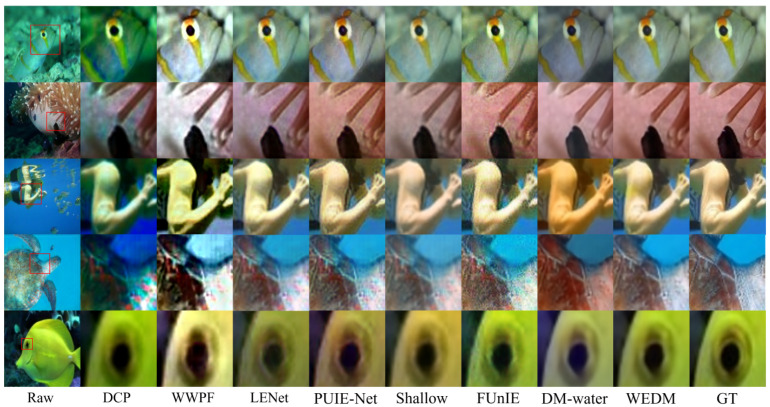
Visual comparison on real underwater images. The red boxes indicate enlarged regions for detail comparison.

Figure 9 compares the local magnification effects of various methods on underwater image enhancement. Key regions (such as fish eyes, tentacles, and sea turtle edges) are annotated and magnified to showcase each method’s performance in detail restoration and color reproduction. When magnified, the original underwater images often exhibit low contrast, noise, blurring, distortion, and color shifts. The DCP method produces a greenish tint after enhancement, while WWPF, despite increasing overall brightness, results in blurred local details and lacks depth. This approach struggles to handle severe degradation in complex scenes, leading to unnatural results. LENet alleviates color distortion but fails in recovering fine details, especially in high-frequency texture areas, where blurring or information loss is common. PUIE-Net performs well in adjusting contrast and brightness, but its limited adaptability to complex underwater environments leads to oversaturation or color shifts in localized areas. Both FUnIE and Shallow methods handle overall color shifts well, but they have clear limitations in detail restoration, often producing blurred edges, artifacts, or color deviations. DM-water, a diffusion-based method, performs better than traditional and CNN-based methods in adapting to lighting and color shifts. However, it underperforms in restoring high-frequency details, leading to blurred edges and introducing unnatural texture artifacts in severely degraded scenes. In contrast, the proposed WEDM method effectively combines low-frequency color compensation with high-frequency detail enhancement through frequency domain decomposition, producing enhanced images that significantly outperform other methods in terms of color accuracy, detail, and visual naturalness, closely resembling the reference images.

#### 4.2.2. Visual Evaluation of Enhancement Results

Underwater images often suffer from diverse and uncontrollable degradations, such as color distortion, low contrast, turbidity, light scattering, and non-uniform illumination. These degradations vary across different scenes, and enhancement methods may behave inconsistently under such variations. Furthermore, common test datasets are often biased toward certain image styles, which may limit the reliability of purely quantitative evaluations.

To better assess the generalization ability of the WEDM across diverse underwater conditions, we uniformly sampled a subset of real-world underwater images from several public datasets, including representative scenarios such as high turbidity, dominant green-blue tones, low lighting, and specular reflections.

**Figure 10:**
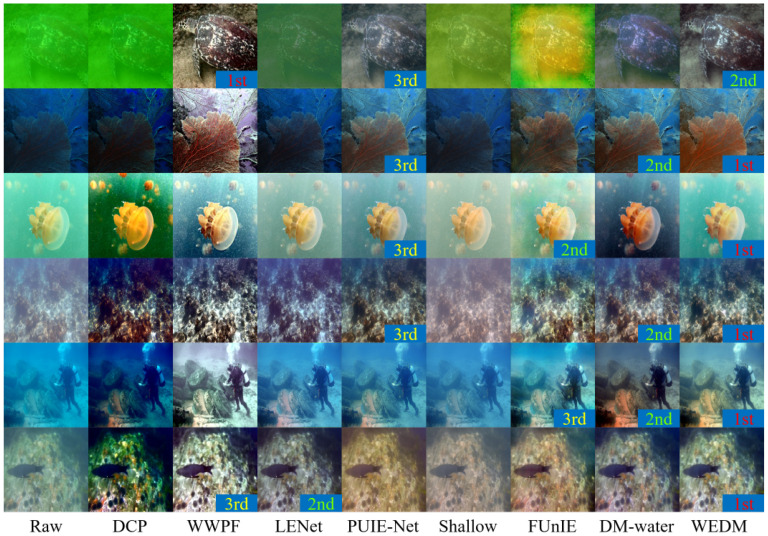
Comparison of image enhancement effects for different styles.

Figure 10 shows six representative examples with enhancement results from different methods. To support perceptual comparison, we ranked the outputs in each row based on visual factors including color accuracy, detail preservation, and naturalness.

As illustrated in Figure 10, the WEDM consistently ranks among the top performers in most scenarios. It produces visually natural outputs with restored colors and clear textures, demonstrating strong adaptability and generalization to various underwater image styles.

### 4.3. Applicability Analysis

**Figure 11:**
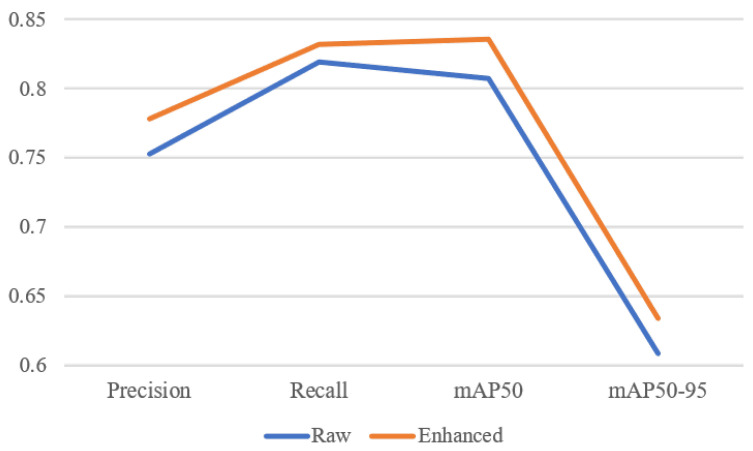
Performance comparison on object detection between raw and enhanced underwater images using YOLOv9.

The enhanced images exhibit improved brightness, contrast, and color fidelity, which are beneficial for high-level vision tasks. These enhancements effectively improve both task adaptability and performance, making them more suitable for underwater vision applications. To verify whether the enhanced images enrich visual features, we conduct object detection experiments on both degraded and enhanced images. The YOLOv9 algorithm is employed to assess the impact of enhancement on detection accuracy. As illustrated in Figure 11, the proposed WEDM framework significantly improves detection performance in terms of precision, recall, and mAP metrics.

**Figure 12:**
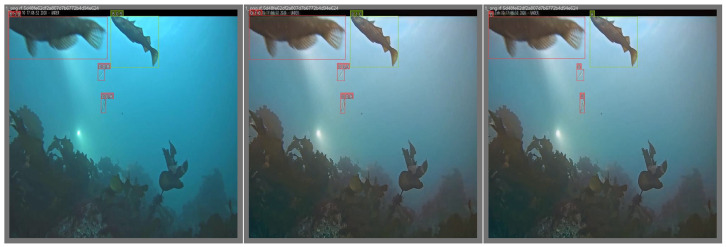
Qualitative comparison of object detection results: (**left**) raw image, (**middle**) enhanced image using the WEDM, and (**right**) ground truth annotations.

Additionally, Figure 12 presents visual comparisons, demonstrating that enhanced images provide clearer edges and more distinguishable features than degraded ones. These improvements confirm the practical effectiveness of our method in real-world underwater detection scenarios.

#### Ablation Tests

To further demonstrate the effectiveness of our proposed method and analyze the impact of each module on underwater image enhancement, we conducted ablation experiments as follows: (1) the Wavelet Color Compensation Module (WCCM) was removed (denoted as w/o WCCM), using only the degraded image *y* as the conditional guide; (2) the Wavelet Convolution Residual Diffusion Adjustment Module (WDM) was removed (denoted as w/o WDM).

**Figure 13:**
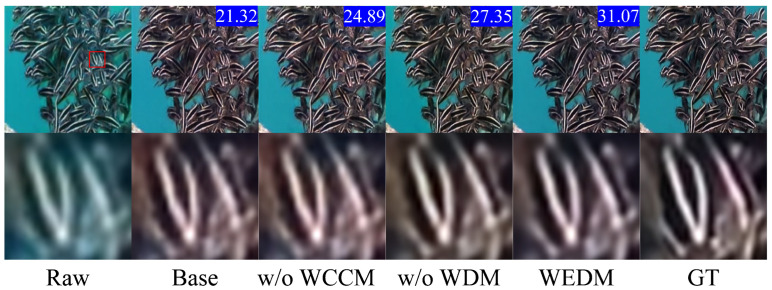
Ablation results comparison. The PSNR metric is indicated in the upper right corner.

Figure 13 shows a visual comparison among the proposed WEDM, the baseline model (Base), the model without the WCCM (w/o WCCM), and the model without the WDM (w/o WDM) in the underwater image restoration task. The GT image represents the ideal, distortion-free underwater scene and serves as the benchmark for restoration quality. Table 3 presents the quantitative results of the ablation experiments on the TEST-L400 dataset.

Based on the results presented in Table 3, we can confirm the effectiveness of both the proposed WCCM and WDM modules, with the WDM achieving the best performance across all metrics. As shown in Figure 13, the baseline model, which uses only the basic diffusion process, exhibits significant color distortion and detail blurring. The model without the WCCM shows some improvement in color correction; however, the recovery of high-frequency details remains insufficient, leading to limited enhancements in contrast and clarity. In contrast, the model without the WDM demonstrates notable color restoration but falls short in recovering details and textures compared to the full WEDM method. Specifically, the WCCM leverages discrete wavelet transform for frequency domain decomposition and enhancement fusion, effectively compensating for color distortion and detail loss in underwater images. Meanwhile, the WDM integrates frequency domain features deeply into the diffusion model via WTConv, further enhancing the restoration of image details and textures. The combination of these modules enables the WEDM to outperform in underwater image restoration tasks, thus further validating the efficacy of the proposed modules in enhancing image quality.

## 5. Conclusions

In this paper, we propose a novel UIE framework, named the WEDM, which effectively combines wavelet-based frequency-domain processing with diffusion models for underwater image enhancement. By fully leveraging the frequency domain characteristics, our Wavelet Color Compensation Module (WCCM) and Wavelet Diffusion Module (WDM) enable precise color correction and efficient frequency-specific feature extraction. The proposed framework, the WEDM, outperforms existing methods on various UIE benchmarks, demonstrating significant improvements in both PSNR and SSIM metrics. Extensive ablation studies confirm the effectiveness of each component, particularly the residual learning strategy, which ensures stable training and robust generalization.

However, due to the use of two diffusion models, our approach does not offer advantages in terms of inference speed. As shown in the results, the WEDM’s computational cost remains higher than recent methods. In the future, we aim to optimize the sampling process and explore lightweight wavelet pruning techniques to enhance the real-time applicability of our framework, while also considering its potential for video-based underwater enhancement and multi-modal sensor fusion applications.

## Figures and Tables

**Table 1:** Comparison of PSNR and SSIM for enhanced results.

Method	TEST-L400		UIEB	
	PSNR	SSIM	PSNR	SSIM
UDCP	14.53	0.656	12.64	0.617
WWPF	18.29	0.759	19.04	0.823
PUIE-Net	28.53	0.917	22.47	0.883
LENet	26.64	0.929	23.37	0.891
Shallow	23.26	0.878	19.45	0.754
FUnIE	23.11	0.823	20.16	0.819
DM-water	29.95	0.946	23.19	0.893
WEDM	35.44	0.961	24.23	0.910

**Table 2:** Comparison of UIQM and UCIQE for enhanced results.

Method	Challenge60		U45		Enhanced Time
	UIQM	UCIQE	UIQM	UCIQE	
UDCP	1.36	0.55	2.30	0.59	1.120 s
WWPF	2.34	0.58	2.80	0.60	0.228 s
PUIE-Net	2.53	0.56	3.15	0.57	0.015 s
LENet	2.58	0.57	3.07	0.59	0.010 s
shallow	2.30	0.50	2.89	0.52	0.050 s
FUnIE	2.37	0.54	3.22	0.58	0.057 s
DM-water	2.56	0.58	2.96	0.60	0.862 s
**WEDM**	2.70	0.59	3.28	0.62	0.110 s

**Table 3:** Metrics of ablation experiments for comparison.

Baselines	PSNR	SSIM
Base	29.12	0.865
w/o WCCM	32.80	0.947
w/o WDM	31.54	0.958
Full model	35.44	0.963

“w/o” refers to the model without the specified module.

## Data Availability

Data sharing applicable.
